# Dynamics of glucose and insulin concentration connected to the *β*‐cell cycle: model development and analysis

**DOI:** 10.1186/1742-4682-9-46

**Published:** 2012-11-19

**Authors:** Martina Gallenberger, Wolfgang zu Castell, Burkhard A Hense, Christina Kuttler

**Affiliations:** 1 Institute of Biomathematics and Biometry, Helmholtz Zentrum München, German Research Center for Environmental Health, Neuherberg, Germany; 2 Scientific Computing Research Unit, Helmholtz Zentrum München, German Research Center for Environmental Health, Neuherberg, Germany; 3 Department of Mathematics, Technical University Munich, Garching, Germany

**Keywords:** Glucose‐insulin regulation, Cell cycle, Feedback loop, ODE model

## Abstract

**Background:**

Diabetes mellitus is a group of metabolic diseases with increased blood glucose concentration as the main symptom. This can be caused by a relative or a total lack of insulin which is produced by the *β*‐cells in the pancreatic islets of Langerhans. Recent experimental results indicate the relevance of the *β*‐cell cycle for the development of diabetes mellitus.

**Methods:**

This paper introduces a mathematical model that connects the dynamics of glucose and insulin concentration with the *β*‐cell cycle. The interplay of glucose, insulin, and *β*‐cell cycle is described with a system of ordinary differential equations. The model and its development will be presented as well as its mathematical analysis. The latter investigates the steady states of the model and their stability.

**Results:**

Our model shows the connection of glucose and insulin concentrations to the *β*‐cell cycle. In this way the important role of glucose as regulator of the cell cycle and the capability of the *β*‐cell mass to adapt to metabolic demands can be presented. Simulations of the model correspond to the qualitative behavior of the glucose‐insulin regulatory system showed in biological experiments.

**Conclusions:**

This work focusses on modeling the physiological situation of the glucose‐insulin regulatory system with a detailed consideration of the *β*‐cell cycle. Furthermore, the presented model allows the simulation of pathological scenarios. Modification of different parameters results in simulation of either type 1 or type 2 diabetes.

## Introduction

The term diabetes mellitus describes a group of metabolic diseases where cells, mainly muscle and fat cells, are not able to take up enough glucose from the blood. This can be due to a relative or absolute lack of insulin (cf. [1,2]). Insulin is the hormone that increases the permeability of the cell membrane for glucose molecules and regulates in this way the uptake of glucose in the cells. Therefore, a lack of insulin leads to a failure of regulation of glucose homeostasis and causes the main symptom of diabetes mellitus, a persisting increased concentration of blood sugar ‐ in technical terms hyperglycemia.

The more common type 2 diabetes ‐ formerly known as adult onset diabetes ‐ is characterized by insulin resistance of the target cells. Type 1 diabetes in contrast is an autoimmune disease where the organism destroys the insulin producing *β*‐cells [3]. In both scenarios glucose‐insulin regulation is disturbed and the adaption of *β*‐cells is insufficient to compensate for this dysfunction.

There are three main players in the glucose‐insulin regulatory system: 

1. Glucose is the energy source for the cells and is mainly obtained by carbohydrates in food. An elevation of blood glucose concentration is detected by the *β*‐cells. It causes them to release stored insulin molecules and to produce new insulin.

2. Insulin is the main regulator of glucose uptake in target cells. It increases the permeability of the cell membrane for glucose molecules.

3. The *β*‐cells are located in the islets of Langerhans in the pancreas. They store and produce insulin.

The following sections describe a mathematical model for the glucose‐insulin regulatory system that connects dynamics in the *β*‐cell with dynamics in the blood and the *β*‐cell cycle. The development of the model is based on the classic insulin secretion model of Grodsky [4], who used a *packet distribution hypothesis* also described by Ličko [5] in greater detail. In these publications insulin is assumed to be stored in packets for different release thresholds of glucose and the main objective is to account for staircase stimulations of glucose. In our work the classic model of Grodsky is extended to variable glucose and adapted to the extension with insulin and glucose blood concentrations and the *β*‐cell cycle. The aim of our model is not to show biochemical or biophysical processes in detail but to present the core processes and interactions in a mechanistic way. The model also provides possibilities for extensions and consideration of additional and more detailed knowledge and questions.

The paper is organized as follows. The motivation of the model, the general setup, and the development are presented in Section “Aim and development of the model”. In Section “Mathematical model” a detailed description of the mathematical model is shown. The mathematical analysis is presented in Section “Analysis of the model” and simulations of the model in Section “Simulation”. The results are summarized and discussed in Section “Discussion”.

## Aim and development of the model

Several publications [6]‐ [8] discuss the relevance of the *β*‐cell mass for the development of diabetes mellitus. Normally there is a slow turnover of *β*‐cells (see [9]) but the *β*‐cell mass can adapt to metabolic demands [7,8]. The concept of dynamic *β*‐cell mass was under discussion for some time but is now generally accepted. Nevertheless, there is a controversy on the mechanisms and the precise growth factors responsible for this adaption [6,9,10]. It was shown that elevated glucose levels enhance *β*‐cell replication [11,12]. More precisely, Porat et al. [13] identify the glucose metabolism via glucokinase as the main positive regulator of *β*‐cell proliferation. As the model in our work does not explicitly account for the glucose metabolism, the more general approach of glucose concentration as regulator of *β*‐cell proliferation is used.

Mathematical models to understand the processes of the glucose‐insulin regulatory system have a long history. Starting with the pioneering work of Bolie [14] in the 1960s one of the first widely used models was the minimal model developed by Bergman and coworkers (see [15,16]) in the beginning of the 1980s. Elaborate reviews of different models using ODE, PDE, DDE, and integro‐differential equations (IDE) are given for example in Makroglou et al. [17] or Boutayeb and Chetouani [18]. In the last decade, several models dealing with the interplay of glucose, insulin, and *β*‐cell mass have been developed. For example see the work of de Winter et al. [19], Topp et al. [20], De Gaetano et al. [21], or the delay‐model of Li et al. [22]. Other models consider particular aspects, as, e.g., electrical activity of *β*‐cells in Cha et al. [23], islet size distribution in Jo et al. [24], or glucose regulation in the whole‐body system in Kang et al. [25]. Other models are designed to control the maintenance of normoglycemia in patients, like the compartment model in [26].

In our approach, based on the results in [6]‐ [11], instead of modeling *β*‐cell mass the whole *β*‐cell cycle is taken into account and plays an important role in the regulatory system. The main aspect of our model is the coupling of insulin storage and of insulin and glucose blood concentrations with the *β*‐cell cycle. It provides the possibility to study precisely the mechanism of glucose influence on the *β*‐cell cycle and therefore on *β*‐cell mass. The model shows the dynamics of glucose and insulin with influence of glucose on the *β*‐cell cycle. Therefore, the dynamics in the blood are directly connected with the mechanisms in the islets of Langerhans.

The model analyzes the interplay of three different negative regulation feedback loops which live on different time scales. With elevated blood glucose concentration insulin release and provision is enhanced which leads to a decrease in glucose levels. Note that the term provision here comprises the generation of insulin, both from stored precursors which might dominate in the beginning and from synthesis of further insulin. 

1. The fastest feedback loop consists in a release of stored insulin immediately after glucose stimulus via elevated blood glucose concentrations [4]. This first insulin peak reaches its maximum after about three to five minutes.

2. The second feedback loop is due to the glucose dependent enhancement of insulin provision. This has a visible effect after about 10 minutes [4].

3. The slowest feedback loop consists of the enhancement of the *β*‐cell cycle by glucose. If the first two reactions of the system are not sufficient to end hyperglycemia, the blood glucose concentration remains at an elevated level. This mild hyperglycemia results in an enhancement of the *β*‐cell cycle leading to more *β*‐cells which in turn can produce further insulin (see [9]).

There are different processes that increase *β*‐cell mass via cell number [8,9]. Besides replication of existing cells, there is also neogenesis by transdifferentiation and stem cells. As an assumption in our paper, based on publications [27]‐ [29], the adaption of *β*‐cell mass is managed by replication only.

**Figure 1:**
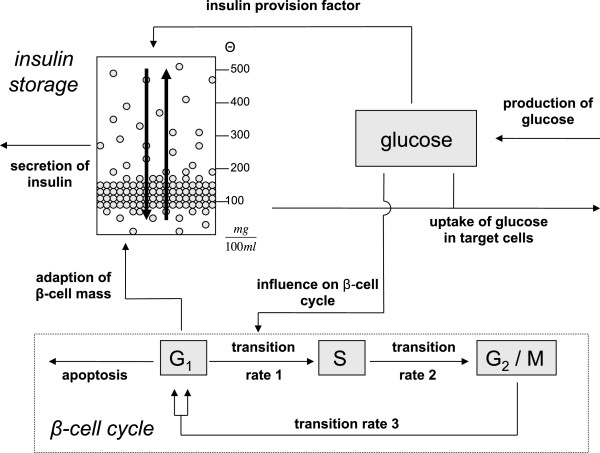
**Schematic concept of the model.** Glucose is given to the system at a constant production rate mainly by the liver. Elevated blood glucose levels lead to immediate release of stored insulin and an enhanced insulin provision. Also, glucose influences the transition rate between phases *G*_1_ and *S* of the cell cycle. Insulin regulates the uptake of glucose in target cells. The molecules are stored in packets with different release thresholds. These packets can be redistributed within the storage.

Figure 1 shows a schematic concept of the model. For simulation of the regulatory system the model is stimulated via elevated blood glucose level.

Besides the uptake of glucose through food, there is also a production of glucose by the organism itself incorporated into the model with a constant production rate. The glucose stimulus induces the organism to release stored insulin and to enhance insulin provision. Insulin is stored in packets for different release thresholds [4]. The storage is filled through glucose dependent insulin provision and cleared at a constant secretion rate. Secreted insulin regulates the uptake of glucose in muscle and fat cells. Besides the immediate release and enhanced provision per cell, the model also accounts for the slowest regulation feedback loop, i.e. glucose influencing the *β*‐cell cycle. The replication of *β*‐cells eventually leads to the provision of more insulin.

The aim of our work is to describe the three different feedback loops in one model and to provide a basis for understanding and explanation of the mechanisms in the glucose‐insulin regulatory system.

The different parts of the model will be described in detail in the following section.

## Mathematical model

### *β*‐cell cycle

The mathematical model consists of three parts where the first one is the *β*‐cell cycle.

The model accounts for three phases of the cell cycle [30]: 

1. The *G*_1_‐phase is a growth phase where the cell prepares for synthesis. A basic assumption of the model is that the functioning *β*‐cell mass lies in this phase [31].

2. The *S*‐phase is the synthesis phase where DNA replicates.

3. The *G*_2_/*M*‐phase is the premitosis and mitosis phase where the nuclear division takes place.

Biological experiments concerning the cell cycle are often done by flow cytometry. This method measures the DNA content in the different phases and can not distinguish between phases *G*_2_ and *M* that have the same DNA content. Therefore, both are combined to one *G*_2_/*M*‐phase.

**Figure 2:**
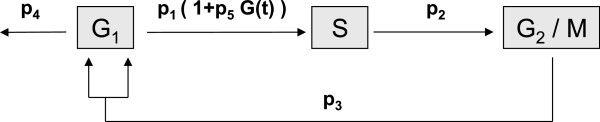
***β*‐cell cycle.** Cell cycle of the *β*‐cells with three phases (*G*_1_, *S*, *G*_2_/*M*) and transition rates (*p*_1_,*p*_2_,*p*_3_). The apoptosis rate is given with parameter *p*_4_. The term *p*_1_(1 + *p*_5_*G*(*t*)) describes a linear influence of glucose on the transition rate *p*_1_ with influence factor *p*_5_.

As it is shown in Figure 2 glucose modifies the transition rate from *G*_1_‐ to *S*‐phase. Several authors claim this transition to be an important checkpoint for the regulation of the *β*‐cell cycle [32]. The linearity of the glucose influence, *p*_1_[1 + *p*_5_*G*], is a simplifying assumption in our work. 

In the model, the transition rates *p*_1_,*p*_2_,*p*_3_, the apoptosis rate *p*_4_, and the influence factor of glucose *p*_5_ are considered. All parameters can be found in Table 1.

**Table 1:** Model parameters

	**Value**	**Definition**
*p*_1_	$6.0594\times 10^{-5}\frac{1}{\min}$	transition rate *G*_1_ → *S*
*p*_2_	$4.9861\times 10^{-3}\frac{1}{\min}$	transition rate *S* → *G*_2_/*M*
*p*_3_	$8.9444\times 10^{-4}\frac{1}{\min}$	transition rate *G*_2_/*M* → *G*_1_
*p*_4_	$3.3194\times 10^{-4}\frac{1}{\min}$	apoptosis rate
*p*_5_	$0.056\frac{100\;\text{ml}}{\text{mg}}$	influence factor of glucose
*p*_6_	0.3 $\frac{\text{mg}}{100\;\text{ml}\;\min}$	rate of glucose production
*p*_7_	0.003 $\frac{1}{\min}$	glucose effectiveness
		at zero insulin
*p*_8_	$360\times 10^{-3}\frac{100\;\text{ml}}{\text{mg min}}$	insulin sensitivity
*p*_9_	0.622 $\frac{1}{\min}$	secretion rate of insulin
*p*_10_	0.3 $\frac{1}{\min}$	decay rate of insulin
*p*_11_	0.0337 $\frac{1}{\min}$	rate of provision increase
*p*_12_	1.72×10^−9^ mg	average insulin amount per *β*‐cell
*bv*	3.33 ml	blood volume of 35g mouse
*f*	0.5	proportionality factor
*h*	5	Hill coefficient
*P*_0_	186.506 $\frac{\text{mg}}{100\;\text{ml}}$	Michaelis constant
${\bar{X}}_{\text{max}}$	1.65×10^−3^ mg	maximal amount of insulin
		per pancreas
*k*	3.3	Hill coefficient
*C*	149.78 $\frac{\text{mg}}{100\;\text{ml}}$	Michaelis constant

Parameters of model (9) used for the simulations shown in Figures 5 and 6 in Section ’Simulation’. The parameter values are based on biological experiments of [4,20,31,32].

An important contributing factor to the *β*‐cell dynamics is glucose toxicity described by Unger et al. [33]. This term refers to the wide range of harmful effects of chronic hyperglycemia leading to chronic oxidative stress after the onset of diabetes, including damages to the pancreatic islet *β*‐cell (cf. [34,35]). As a consequence of hyperglycemia lipid toxicity may additionally damage *β*‐cells. This effect is called *glucolipotoxicity* and is described in [36]. To account for the effects of glucose toxicity and glucolipotoxicity on insulin secretion a glucose dependent apoptosis rate can be incorporated to the cell cycle. In the actual version of the model the role of glucose toxicity is omitted for the sake of simplicity and the apoptosis rate is constant. As an assumption in the *β*‐cell cycle model, apoptosis and the only under some pathological conditions relevant necrosis are subsumed in the rate *p*_4_ called apoptosis rate [37].

The *β*‐cell cycle is modeled as a three compartment model as it is common in cell cycle modeling (cf. [38]):

(1) $$\begin{array}{lll} {\;Ġ}_{1}(t) & =2\;p_{3}G_{2}/M(t)-\left[p_{1}\left[1+p_{5}G(t)\right]+p_{4}\right]G_{1}(t),\; & \;\\ \;\dot{S}(t) & =p_{1}\left[1+p_{5}G(t)\right]G_{1}(t)-p_{2}S(t),\;\\ \dot{G_{2}/M}(t) & =p_{2}S(t)-p_{3}G_{2}/M(t).\; & \; \end{array}$$

The constant 2 in the first equation accounts for cell division in the transition from *G*_2_/*M*‐ to *G*_1_‐phase. In the physiological case for adults the *β*‐cell cycle is very slow (see [9]) but has the capability of dynamic adaption to metabolic demands [7]. In model (1) glucose influences the transition rate *p*_1_ from *G*_1_‐ to *S*‐phase and is able to regulate the *β*‐cell cycle. This is the case if glucose triggers the system and neither the immediate release of stored insulin nor the enhanced insulin provision is able to lower blood glucose concentration. Then a high level of glucose forces the cell cycle to accelerate.

### Glucose and insulin concentration in the blood

The dynamics of blood glucose concentration are based on the model of Topp et al. [20]. There, the change in blood glucose concentration is modeled as the difference between production and uptake of glucose,

(2) $$Ġ(t)=\text{production}-\text{uptake}=p_{6}-\left[p_{7}+p_{8}I(t)\right]G(t).$$

The net rate of glucose production is represented by a constant production rate *p*_6_. This rate is the difference of an intrinsic glucose production, mainly by the liver, and glucose concentration independent uptake of glucose. The latter consists mainly of glucose uptake by the brain and other nervous tissues which is assumed to be constant in our model. The uptake of glucose in other tissues consists of two processes dependent on the glucose blood concentration. One is an insulin independent uptake represented by the parameter of glucose effectiveness *p*_7_. The other process is an insulin dependent glucose uptake mainly by muscle and fat cells which is influenced by insulin sensitivity *p*_8_ and depends on blood insulin concentration *I*. The parameters are listed in Table 1.

Similarly, the dynamics of blood insulin concentration are modeled as secretion minus degradation,

(3) $$İ(t)=\frac{1}{\mathrm{bv}}\;p_{9}X_{1}(t)-p_{10}I(t),$$

where secretion consists of the secreted amount of insulin molecules from the *β*‐cells, *p*_9_*X*_1_, that has to be considered with respect to the blood volume *bv* of the organism. Variable *X*_1_ will be discussed in detail in the following section. Degradation of insulin is modeled with a constant decay rate *p*_10_.

### Insulin storage

In 1972, Grodsky published a packet distribution hypothesis for insulin granules in an insulin storage [4]. In this work the storage is modeled with no dynamic connection to the remaining glucose‐insulin regulatory system. For stimulation of the system, different glucose functions were considered, for example single‐ or two‐step constant stimulation, staircase stimulation, or ramp functions of glucose concentration.

In our approach the model of Grodsky is incorporated into a model of the glucose‐insulin regulatory system including the adaption of *β*‐cell mass to metabolic demands. Although the publication of this insulin secretion model is several years ago, the packet distribution hypothesis still finds application, for example in the work of Overgaard et al. [39]. There, it is included into a mathematical model for insulin secretion applied to IVGTT and OGTT data. Also Pedersen et al. presented an updated version of the hypothesis for oral minimal models of insulin secretion in [40] and Tsaneva‐Atanasova described insulin secretion in a general context of mechanisms of cell secretion [41].

As the knowledge about *β*‐cell biology has increased since 1972 some reinterpretation of the assumptions in [4] are appropriate. In the original work there is no clear definition of what the packets are. They could be interpreted as insulin containing granules within a cell with different sensitivities for glucose induced release. Although granules clearly are present and variability of sensors for signals is a common feature in biology, there is no direct experimental proof, yet, to our knowledge. An alternative interpretation would be variability of sensitivity on cell level. In fact, Jonkers and Henquin [42] show a sigmoidal distribution of active *β*‐cells. A combination of both aspects (and possibly more unknown factors) may be the most probable explanation for the experimentally found dose‐response curves [43]. Therefore, in the following the packets are interpreted as pancreatic *β*‐cells that can be active or inactive. Several recent models for insulin secretion considering Ca^2 + ^‐evoked exocytosis, the cAMP amplifying pathway, and actual results on granule dynamics are available (see e.g. [44]‐ [46]) but for the qualitative conclusions in our work Grodsky’s basic model of insulin secretion is sufficient.

**Figure 3:**
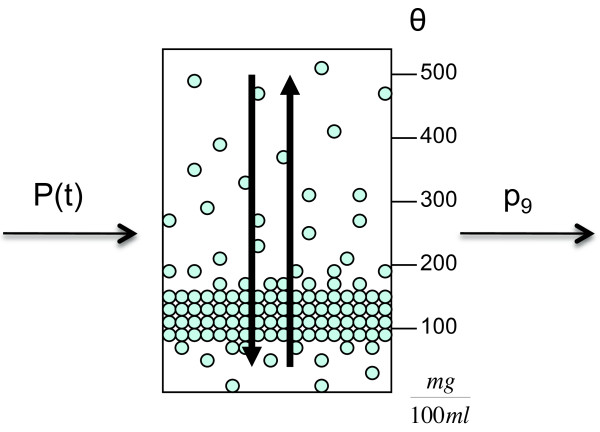
**Insulin storage and packet distribution.** The storage is filled through glucose dependent insulin provision *P*(*t*) and cleared at a constant rate *p*_9_. Insulin is stored in packets at different release thresholds *θ*. A special characteristic of the storage is the redistribution process of packets shown by the vertical arrows. The figure is adapted from [4].

Our first modification of the insulin secretion model [4] is the adaption to time dependent glucose concentrations. In doing so, it obtains a wider field of application and gets connectable to the dynamic regulatory system. In a second step the insulin storage is incorporated with blood glucose and insulin concentrations as well as with the *β*‐cell cycle to form a complete model. It is assumed that insulin is stored in homogeneous packets in the storage which is shown in Figure 3. There are different release thresholds *θ*(in units of glucose concentration) for the packets and the major part of them is stored at lower values of glucose concentration, between 100 and 200 $\;\frac{\text{mg}}{\text{100ml}}$. These lower values are often reached in an organism so that there is the need for sufficient insulin to react to these impulses. 

The initial packet distribution *ξ*(*θ*,0) for threshold *θ* at time *t*=0 is crucial for the storage model. In every time step the different processes within the storage try to achieve this initial distribution at least qualitatively.

The storage is filled through a glucose dependent insulin provision factor *P*, and insulin is released with constant secretion rate *p*_9_. Besides that, there is a redistribution process where packets that were not needed so far are redistributed to qualitatively reestablish the initial packet distribution *ξ*(*θ*,0).

Using the packet distribution the storage can be divided into two compartments. The releasable amount of insulin *X*_1_ contains the packets with threshold value *θ* below the actual blood glucose concentration *G*, i.e.,

$$X_{1}(t)=\overset{G(t)}{\underset{0}{\int }}\xi (\theta ,t)\;\mathrm{d\theta .}$$

These packets can be released from the *β*‐cell for glucose concentration G. The second compartment in the storage pool is the non‐releasable amount of insulin *X*_2_ of the packets with threshold value *θ* as it is dependent on the maximum amount of insulin peabove the actual blood glucose concentration *G*, i.e.,

$$X_{2}(t)=\overset{\infty }{\underset{G(t)}{\int }}\xi (\theta ,t)\;\mathrm{d\theta .}$$

These packets can not be released for glucose concentration G but are involved in the redistribution process.

As, in our work, the insulin storage is connected to the *β*‐cell cycle, changes in the insulin producing *β*‐cell mass influence also the initial distribution *ξ*(*θ*,0) as it is dependent on the maximum amount of insulin per pancreas, ${\bar{X}}_{\text{max}}$. The insulin producing *β*‐cell mass can change in every time step *t* and thus ${\bar{X}}_{\text{max}}$ is time dependent, too. Therefore, this distribution is called target distribution and is denoted by *ξ*^∗^(*θ*,*t*). Consequences of this modification are shown later in this section.

The insulin storage is modeled as a three compartment model with *X*_1_, *X*_2_, and an equation for the dynamics of the provision factor *P*. A detailed version shows the different processes within the storage according to the target distribution:

(4) $$\begin{array}{cccc} {\dot{X}}_{1}(t) & = & \frac{X_{1}(t)}{\int_{0}^{G(t)}\xi^{*}(\theta ,t)\text{dθ}}\xi^{*}(G(t),t)Ġ(t)-p_{9}X_{1}(t)\;\\ & & +\tilde{f}\overset{G(t)}{\underset{0}{\int }}\xi^{*}(\theta ,t)\text{dθ}\left[X_{1}(t)+X_{2}(t)\right]-\tilde{f}\overset{\infty }{\underset{0}{\int }}\xi^{*}(\theta ,t)\text{dθ}X_{1}(t)+f\overset{G(t)}{\underset{0}{\int }}\xi^{*}(\theta ,t)\mathrm{d\theta P}(t),\; & \;\\ {\dot{X}}_{2}(t) & = & \frac{-\;X_{2}(t)}{\int_{G(t)}^{\infty }\xi^{*}(\theta ,t)\text{dθ}}\xi^{*}(G(t),t)Ġ(t)\;\\ & & +\tilde{f}\overset{\infty }{\underset{G(t)}{\int }}\xi^{*}(\theta ,t)\text{dθ}\left[X_{1}(t)+X_{2}(t)\right]-\tilde{f}\overset{\infty }{\underset{0}{\int }}\xi^{*}(\theta ,t)\text{dθ}X_{2}(t)+f\overset{\infty }{\underset{G(t)}{\int }}\xi^{*}(\theta ,t)\mathrm{d\theta P}(t),\; & \;\\ \;\dot{P}(t) & = & p_{11}\left[P_{\infty }(t)-P(t)\right]. \end{array}$$

**Figure 4:**
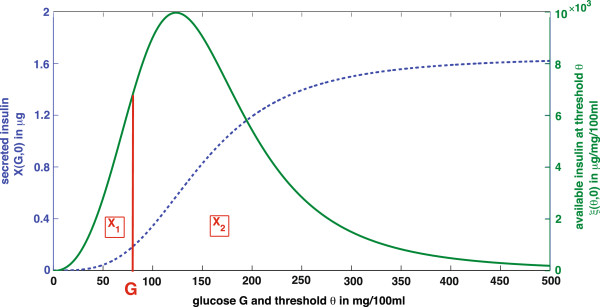
**Initial distribution in the storage and compartments*X***_**1**_**and*X***_**2**_**.** Secreted insulin *X*(*G*,0)(dashed line) is the whole amount of insulin releasable by the pancreas at glucose concentration *G*. It is the integral over the initial packet distribution *ξ*(*θ*,0) (solid line). *X*_1_is the releasable and *X*_2_the non‐releasable amount of insulin. In the plot they can be identified as areas under the solid curve *ξ*(*θ*,0)ranging from *θ* = [0,*G*and from *θ* = *G∞*), respectively. The figure is adapted from [4].

For details concerning the derivation of equations (4) see the Appendix “Derivation of glucose dependent insulin storage dynamics” section. The first terms in the equations for ${\dot{X}}_{1}$ and ${\dot{X}}_{2}$ correspond to the time dependence of the integral limits (chain rule) which is an expansion of the original insulin secretion model. They describe the influence of changing glucose concentration on the separation of the insulin storage into compartments *X*_1_ and *X*_2_ (see Figure 4). This influence depends on the actual glucose concentration value and the change in glucose concentration with time *t*.

System (4) shows the production and redistribution process in detail according to the target distribution *ξ*^∗^(*θ*,*t*). Their contribution to the dynamics of the single compartments are modeled with proportionality factors *f* and $\tilde{f}$, respectively.

To simplify the representation the integral functions are expressed in terms of general transition functions. The simplified version of the model is given in the following:

(5) $$\begin{array}{ccc} {\dot{X}}_{1}(t) & = & \left[\frac{\xi^{*}(G(t),t)Ġ(t)}{{\tilde{f}}^{-1}u_{2}(t)}-u_{1}\left(t\right)-p_{9}\right]X_{1}(t)+u_{2}(t)X_{2}(t)+u_{3}(t)G_{1}(t)P(t),\\ {\dot{X}}_{2}(t) & = & u_{1}(t)X_{1}(t)-\left[\frac{\xi^{*}(G(t),t)Ġ(t)}{{\tilde{f}}^{-1}u_{1}\left(t\right)}+u_{2}\left(t\right)\right]X_{2}(t)+u_{4}(t)G_{1}(t)P(t),\\ \;\dot{P}(t) & = & p_{11}[P_{\infty }(t)-P(t)]. \end{array}$$

The parameters of the model are given in Table 1. The glucose dependent transition functions *u*_1_,…,*u*_4_ describe provision and redistribution processes according to the target distribution. They are given in greater detail in Appendix “Transition functions” section.

The function *P*_*∞*_models the glucose dependent steady state of insulin provision. To express the delay in the effect of insulin provision, *P*_*∞*_ is modeled as a Hill function

$$P_{\infty }\left(t\right)=\frac{G{\left(t\right)}^{h}}{P_{0}^{h}+G{\left(t\right)}^{h}}\;,$$

with *h* being the Hill coefficient and *P*_0_ the Michaelis constant.

An example for a possible initial distribution in case of constant glucose is given in [4]. For constant glucose concentration *G* experimental results [47,48] show the following course of insulin release *X*(*G*,0) in the early phase (dashed line in Figure 4). This evolution is described with a sigmoid function

$$X\left(G,0\right)={\bar{X}}_{\text{max}}\frac{G^{k}}{C^{k}+G^{k}}\;,$$

where ${\bar{X}}_{\text{max}}$ is the maximum amount of insulin per pancreas, *k* the Hill coefficient, and *C* the Michaelis constant. With *X*(*G*,0) being the amount of released insulin at constant glucose concentration *G*, the initial distribution *ξ*(*θ*,0) is expressed as the derivative of *X*(*G*, 0). To be precise,

(6) $$X(G,0)={\bar{X}}_{\text{max}}\frac{G^{k}}{C^{k}+G^{k}}=\overset{G}{\underset{0}{\int }}\xi (\theta ,0)\text{dθ}$$

(7) $$\Rightarrow \;\xi (\theta ,0)=\frac{d}{\text{dθ}}X(\theta ,0)={\bar{X}}_{\text{max}}\frac{kC^{k}\theta^{k-1}}{{\left(C^{k}+\theta^{k}\right)}^{2}}\;.$$

These expressions change in the case of time dependent glucose and in connection with the *β*‐cell cycle that results in variable *β*‐cell mass. First, the maximum amount of insulin per pancreas ${\bar{X}}_{\text{max}}$ is not constant anymore but depends on the *β*‐cell mass *G*_1_ at time *t*,

(8) $$X_{\text{max}}(t)=p_{12}G_{1}(t).$$

The parameter *p*_12_ is interpreted as the amount of insulin per *β*‐cell. One of several possibilities to determine this parameter is the quotient of the maximum amount of insulin per pancreas and the initial amount of *β*‐cells, i.e.,

$$p_{12}=\frac{{\bar{X}}_{\text{max}}}{G_{1}(0)},$$

where *G*_1_ (0) serves as a normalization. Then equation (8) determines for every amount of *β*‐cells *G*_1_ at time *t* > 0 the corresponding maximum amount of insulin. Therefore, expressions (7) and (6) now read as

$$\begin{array}{lll} X^{*}(G(t),t) & =X_{\text{max}}(t)\frac{G{\left(t\right)}^{k}}{C^{k}+G{\left(t\right)}^{k}}=\overset{G(t)}{\underset{0}{\int }}\xi^{*}(\theta ,t)\text{dθ}\; & \;\\ \Rightarrow \;\xi^{*}(\theta ,t) & =\frac{d}{\text{dθ}}X^{*}(\theta ,t)=X_{\text{max}}(t)\frac{kC^{k}\theta^{k-1}}{{\left(C^{k}+\theta^{k}\right)}^{2}}\;,\; & \; \end{array}$$

where *ξ*^∗^(*θ*,*t*) is now called target distribution.

### Complete model

In the previous subsections the partial models have been developed and explained in detail. Based on this outline the complete model can now be formulated:

(9) $$\begin{array}{ccc} \;{Ġ}_{1}(t) & = & 2p_{3}G_{2}/M(t)-\left[p_{1}\left[1+p_{5}G(t)\right]+p_{4}\right]G_{1}(t),\;\\ \;\dot{S}(t) & = & p_{1}\left[1+p_{5}G(t)\right]G_{1}(t)-p_{2}S(t),\;\\ \dot{G_{2}/M}(t) & = & p_{2}S(t)-p_{3}G_{2}/M(t),\;\\ \;Ġ(t) & = & p_{6}-\left[p_{7}+p_{8}I(t)\right]G(t),\\ \;İ(t) & = & \frac{1}{\mathrm{bv}}p_{9}X_{1}(t)-p_{10}I(t),\;\\ \;{\dot{X}}_{1}(t) & = & \left[\frac{\xi^{*}(G(t),t)Ġ(t)}{{\tilde{f}}^{-1}u_{2}\left(t\right)}-u_{1}\left(t\right)-p_{9}\right]X_{1}(t)+u_{2}(t)X_{2}(t)+u_{3}(t)G_{1}(t)P(t),\\ \;{\dot{X}}_{2}(t) & = & u_{1}(t)X_{1}(t)-\left[\frac{\xi^{*}(G(t),t)Ġ(t)}{{\tilde{f}}^{-1}u_{2}\left(t\right)}+u_{2}\left(t\right)\right]X_{2}(t)+u_{4}(t)G_{1}(t)P(t),\\ \;\dot{P}(t) & = & p_{11}\left[P_{\infty }(t)-P(t)\right]. \end{array}$$

The first three equations describe the dynamics of the *β*‐cell cycle with its three phases. The fourth and the fifth equation show the dynamics of blood glucose and insulin concentration, and the last three equations present the insulin secretion model. The partial models are connected through several entities. 

1. Insulin *I* influences the glucose dynamics via insulin dependent uptake in target cells. Secretion of insulin consists of the releasable amount of insulin molecules *p*_9_*X*_1_ in relation to the blood volume *bv*.

2. Glucose *G* plays an important role in regulating the processes within the insulin secretion model. It regulates the provision of insulin and defines the compartments of the insulin storage via the target distribution. It also contributes to the redistribution process. Furthermore, glucose regulates the *β*‐cell cycle via the glucose dependent transition rate from *G*_1_‐ to *S*‐phase.

3. The *β*‐cell mass *G*_1_ determines the capacity of insulin provision.

In Section “Simulation” the behavior of solutions of the complete model can be seen. There, the model is simulated in the physiological case as well as in an experimental situation.

## Analysis of the model

In this section a basic mathematical analysis is presented to achieve a better understanding of the model behavior. First, positivity of the solution is shown. Then the analysis focusses on steady states and their stability to explain the asymptotic development of the solution.

### Positivity of solutions

Our model of the glucose‐insulin regulatory system describes the dynamics of biological quantities, e.g., cell numbers, concentrations, and mass. Naturally, these quantities are positive and therefore positivity of the solution is a desired characteristic of the system. A necessary and sufficient condition for the existence of positive solutions is given in the following corollary.

#### Corollary 1

( [49]): For every *t*_0_ > 0 and initial value $x_{0}=(x_{1}^{0},\ldots ,x_{m}^{0})\in R_{+}^{m}=\{x\in R^{m}:x_{i}>0,i=1,\mathrm{..},m\}$, each component *x*_*i*_, *i* = 1,…,*m*, of the solutions to

$${\dot{x}}_{i}(t)=f_{i}(t,x_{1},\ldots ,x_{m}),\;x_{i}(t_{0})=x_{i}^{0}$$

is positive if and only if

(10) $$f_{i}(t,x_{1},\ldots x_{i-1},0,x_{i+1},\ldots ,x_{m})>0,$$

i = 1,…,m, for all *t* > 0 and $x\in R_{+}^{m}$.

Using the condition from Corollary 1, positivity of the solution of system (9) can be analyzed. The parameters of the model and the initial values are non‐negative (see Tables 1 and 2, respectively). Therefore, it can be shown that condition (10) holds for all positive values of the variables. Summarizing, positivity of the solution of the presented model for the glucose‐insulin regulatory system is guaranteed.

**Table 2:** Initial values

**Variable**	**Initial Value**	**Definition**
*G*_1_(0)	958000	number of cells in *G*_1_‐phase
*S*(0)	14000	number of cells in *S*‐phase
*G*_2_/*M*(0)	28000	number of cells in *G*_2_/*M*‐phase
*G*(0)	200 $\frac{\text{mg}}{100\;\text{ml}}$	blood glucose concentration
*I*(0)	0.01 $\frac{\text{mg}}{100\;\text{ml}}$	blood insulin concentration
*X*_1_(0)	0.0012 mg	releasable amount of insulin
*X*_2_(0)	0.0005 mg	non‐releasable amount of insulin
*P*(0)	0	provision of insulin

**Figure 5:**
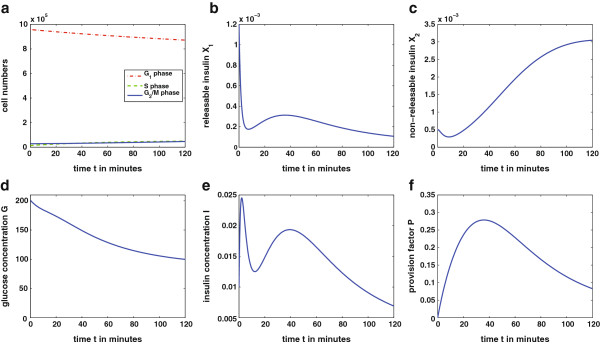
**Solution of the complete model over 120 minutes.** The simulation presents the physiological case of the glucose‐insulin regulatory system over 120 minutes after a high initial glucose value. The initial values for this simulation (with corresponding units) are given in Table 2 and the parameters in Table 1. The solution of the complete model (9) was achieved numerically using Matlab ODE45.

**Figure 6:**
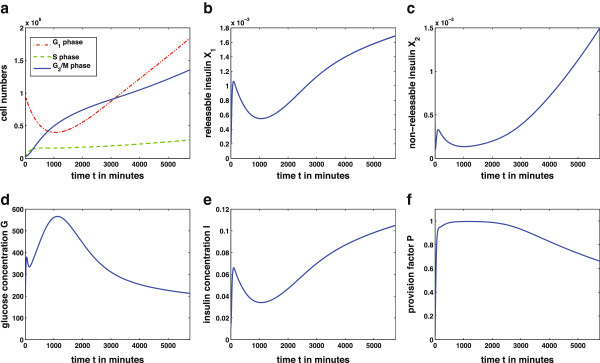
**Solution of the complete model over 96 hours.** The simulation presents an experimental situation with high glucose infusion over 96h. The parameter *p*_6_ of glucose production was increased up to ${\tilde{p}}_{6}=8.6806\frac{\text{mg}}{100\;\text{ml}\;\min}$. The *β*‐cell mass increases due to the persisting hyperglycemia. With the adaption of *β*‐ cell mass, seen in Figure 6a) the glucose‐insulin regulatory system is able to reach euglycemia, i.e. the stable steady state $F_{2}^{*}$. Other parameters and initial values for this simulation are given in Tables 1 and 2, respectively. The solution of the complete model (9) was achieved numerically using Matlab ODE45.

Initial values and definition of variables in model (9) used for the simulations shown in Figures 5 and 6.

### Steady states

As it can be seen in Section ‘Simulation’, the graph of the solution suggests a steady state behavior of the system. The investigation of the steady states is based on the *β*‐cell cycle model (1).

We determined the only two steady states for the *β*‐cell cycle. There is a trivial steady state where no cells are present,

(11) $$G_{1}^{*}=S^{*}=G_{2}/M^{*}=0,$$

and there is another steady state where the number of apoptotic cells equals the number of new cells. This occurs if the apoptosis rate equals the transition rate from *G*_1_‐ to *S*‐phase:

(12) $$p_{4}=p_{1}(1+p_{5}\hat{\text{G}})\;\Leftrightarrow \;\hat{\text{G}}=\frac{p_{4}-p_{1}}{p_{1}p_{5}}.$$

Equation (12) results in a fixed value for glucose, $\hat{\text{G}}$, that can be modified via the influence factor *p*_5_. $\hat{\text{G}}$ is a crucial threshold for the development of the cell cycle as it determines the values of glucose concentration leading to *β*‐cell mass increase or decrease.

The apoptosis rate *p*_4_ is greater than the transition rate from *G*_1_‐ to *S*‐phase if the actual value of glucose concentration *G*_*t*_ is lower than $\hat{\text{G}}$:

(13) $$p_{4}>p_{1}(1+p_{5}G_{t})\;\Leftrightarrow \;\hat{\text{G}}>G_{t}.$$

In this case there are more *β*‐cells dying than dividing per time step and in consequence the total amount of *β*‐cells in *G*_1_‐phase is decreasing.

In contrast, the apoptosis rate *p*_4_ is lower than the transition rate from *G*_1_‐ to *S*‐phase if the actual value of glucose concentration *G*_*t*_ is greater than $\hat{\text{G}}$:

(14) $$p_{4}<p_{1}(1+p_{5}G_{t})\;\Leftrightarrow \;\hat{\text{G}}<G_{t}.$$

Fewer *β*‐cells are dying than dividing per time step and in consequence the *β*‐cell mass in *G*_1_‐phase is increasing.

In summary, the further analysis of the steady state behavior results in only two fixed points for positive values of the variables. The two steady states (11) and (12) of the cell cycle model determine the two steady states of the whole model. The first one is the trivial steady state

(15) $$F_{1}^{*}=\left(0,0,0,\frac{p_{6}}{p_{7}},0,0,0,P_{\infty }\left(\frac{p_{6}}{p_{7}}\right)\right)$$

with all cell numbers in the three phases equal to zero. As it can be seen in $F_{1}^{*}$, glucose concentration is never equal to zero due to constant production of glucose by the liver. Thus, the steady state of the provision factor, *P*_*∞*_, is also not equal to zero but a fixed value depending on $G^{*}=\frac{p_{6}}{p_{7}}$.

The second steady state is driven by the threshold value $\hat{\text{G}}$ determining a non‐trivial steady state

(16) $$F_{2}^{*}=\left(G_{1}^{*},S^{*},G_{2}/M^{*},G^{*},I^{*},X_{1}^{*},X_{2}^{*},P^{*}\right).$$

The values for the different variables can be given explicitly:

$$\begin{array}{ccc} \;G_{1}^{*} & = & \frac{p_{9}X_{1}^{*}}{p_{12}fP^{*}},\;S^{*}=\frac{p_{4}p_{9}X_{1}^{*}}{p_{2}p_{12}fP^{*}},\;G_{2}/M^{*}=\frac{p_{2}}{p_{3}}S^{*}=\frac{p_{4}p_{9}X_{1}^{*}}{p_{3}p_{12}fP^{*}},\;\\ \;G^{*} & = & \hat{\text{G}}=\frac{p_{4}-p_{1}}{p_{1}p_{5}},\;I^{*}=\frac{p_{6}-p_{7}G^{*}}{p_{8}G^{*}},\;\\ \;X_{1}^{*} & = & \frac{\mathrm{bv}p_{10}I^{*}}{p_{9}},\;X_{2}^{*}=\frac{X_{1}^{*}}{f}\left[p_{9}\;\left[\;1+{G^{*}}^{-h}P_{0}^{h}\;\right]-f\right],\;P^{*}=\frac{{G^{*}}^{h}}{P_{0}^{h}+{G^{*}}^{h}}.\; \end{array}$$

The steady state $F_{2}^{*}$ is reached after a glucose stimulus to the regulatory system. The variables tend to these values if no further impulse or modification to the system is following.

### Stability

The stability of the two steady states $F_{1}^{*}$ and $F_{2}^{*}$ can be investigated by computing the Jacobian matrix of these fixed points. This analysis is based on the system parameters in Table 1.

In summary, it can be shown that there are two types of steady states for the system. 

1. The trivial steady state with cell numbers equal to zero,

$$F_{1}^{*}=\left(0,0,0,\frac{p_{6}}{p_{7}},0,0,0,P_{\infty }\left(\frac{p_{6}}{p_{7}}\right)\right),$$

is an unstable fixed point.

2. The non‐trivial steady state,

$$F_{2}^{*}=\left(G_{1}^{*},S^{*},G_{2}/M^{*},G^{*},I^{*},X_{1}^{*},X_{2}^{*},P^{*}\right),$$

is a stable fixed point reached by the system after some time without any influence from the outside.

The behavior of the model according to the mathematical analysis will be illustrated in the following section using simulations of the complete model.

## Simulation

In this section two simulations of the complete model (9) are presented. The first simulation describes the behavior of the glucose‐insulin regulatory system in the physiological case. The second simulation particularly shows the adaption of *β*‐cell mass under long term glucose infusion [12].

The physiological case ‐ given in Figure 5 ‐ has been simulated over 120 minutes with a high initial glucose value resulting for example from recent food intake. Parameters and initial values of the variables are given in Tables 1 and 2, respectively. The following description discusses the subplots. 

• **Figure**5**a:** With the given parameters the threshold value for the cell cycle is $\hat{\text{G}}=80\frac{\text{mg}}{100\;\text{ml}}$. Glucose concentration *G* is above this level for 120 minutes. Therefore, the cell cycle reacts in the following way: Glucose values above the threshold increase the transition rate from *G*_1_‐ to *S*‐phase. As the *β*‐cell cycle is a slow process, in the first 120 minutes an only slight increase in *S*‐ and *G*_2_/*M*‐phase is detectable while the cell number in *G*_1_‐phase is decreasing. Increase in *β*‐cell mass, i.e., *G*_1_, takes more than 120 minutes. As glucose concentration is almost at the steady state $\hat{\text{G}}=80\frac{\text{mg}}{100\;\text{ml}}$ towards the end of the simulation, the system will regulate itself without significant adaption of *β*‐cell mass. This is expected in the physiological case without abnormal exposure to glucose.

• **Figure**5**b:** The releasable amount of insulin *X*_1_ shows a biphasic behavior. There is a first peak release of stored insulin molecules and a second phase in consequence of provision of further insulin.

• **Figure**5**c:** With decreasing glucose concentration there are more packets with threshold value above the actual glucose level. For this reason and due to enhanced insulin provision *P* the amount of non‐releasable insulin *X*_2_ increases.

• **Figure**5**d:** Glucose concentration *G* is decreasing from the high initial value as there is an increased concentration of insulin *I* in the blood.

• **Figure**5**e:** Blood insulin concentration *I* follows with some delay the releasable amount of insulin. It also shows the characteristic biphasic behavior of insulin release.

• **Figure**5**f:** Provision factor *P* shows an increase in presence of high glucose values and decreases as blood glucose decreases.

The second simulation is done according to the experiments of Bonner‐Weir et al. [12]. There, rats are given a high glucose infusion for 96 hours. After this time a significant increase in *β*‐cell mass is observable. The design of the experiment is assigned to the mathematical model (9) in the following way: the glucose production rate was increased from $p_{6}=0.3\frac{\text{mg}}{100\;\text{ml}\min}$ up to ${\tilde{p}}_{6}=8.6806\frac{\text{mg}}{100\;\text{ml}\;\min}$ to account for a high concentrated glucose infusion. The resulting plots of the solution of model (9) are shown in Figure 6. Most important, a significant increase of *β*‐cells *G*_1_can be seen in Figure 6a. This results from the persisting and severe hyperglycemia (Figure 6d) due to the high glucose production ${\tilde{p}}_{6}$. With longer time of simulation the system will reach the stable steady state $F_{2}^{*}$. The transfer of the experimental design in [12] to our model shows qualitatively that the glucose‐insulin regulatory system is able to achieve euglycemia through adaption of *β*‐cell mass as it is stated in several publications (e.g. [6]‐ [8,13]). Note that the model currently assigns increase of *β*‐cell biomass exclusively to cell number (hyperplasia). It disregards an increase in cell size (hypertrophy) which occurs in [12]. This simplification which can be overcome in further development of the model leads to an overestimation of changes in cell division rate as reflected in Figure 6a. Probably especially the first responses to elevated glucose concentrations are concerned. 

## Discussion

This work presents a mathematical model that describes three different negative regulation feedback loops of the glucose‐insulin regulatory system: 

1. Immediate release of stored insulin molecules.

2. Enhancement of provision of new insulin.

3. Adaption of the *β*‐cell cycle to metabolic demands.

This is possible by incorporating an insulin secretion model describing storage and release of insulin molecules on the one hand and insulin provision on the other hand [4]. Furthermore, insulin provision is glucose dependent which allows its adaption to specific demands of the organism at every time step. The third feedback loop is modeled via incorporation of the *β*‐cell cycle with glucose regulating the replication rate of the cells [11,13].

Several models of the glucose‐insulin regulatory system, as e.g., [19]‐ [21], describe glucose, insulin and *β*‐cell mass dynamics, whereas our model shows the connection of glucose, and insulin concentrations with the *β*‐cell cycle as the main aspect. In this way the important role of glucose as regulator of the cell cycle [13] and the capability of the *β*‐cell mass to adapt to metabolic demands can be analyzed in detail. Hereby, the adaption of *β*‐cell mass is assigned exclusively to hyperplasia and disregards hypertrophy.

The model conserves typical characteristics of the glucose‐insulin regulatory system. The plots of the complete model in Figure 5 show biphasic insulin release represented through the biphasic shape of the releasable amount of insulin *X*_1_. This is a typical behavior of insulin release reported in several biological publications (e.g. [47,50]).

Modeling insulin secretion based on [4] incorporates three feedback loops consisting of stored insulin, provision of further insulin, and variable *β*‐cell mass. Our model expands classic insulin secretion models (e.g. [4,44]‐ [46]) by a connection to the *β*‐cell cycle.

The qualitative behavior of the model is illustrated with simulations. In the physiological case, shown in Figure 5, the *β*‐cell mass is sufficient to produce and release enough insulin to decrease glucose concentration and maintain euglycemia. With a second simulation the adaption of the *β*‐cell mass to increasing metabolic demands is presented. This situation occurs in long term studies with persisting hyperglycemia as it can be seen in Figure 6. In a simulation similar to the experiment in [12] increase of *β*‐cell mass via hyperplasia in 96 hours of hyperglycemia is shown.

The analysis of the model gives the existence of two steady states. One describes a trivial fixed point with *β*‐cell numbers in all three phases equal to zero. The second steady state is a stable fixed point resulting from successfully achieving euglycemia. The trivial steady state is unstable while the stable non‐trivial steady state will be attained in both situations shown in Figures 5 and 6 with longer simulation times. The stability of the steady states is dependent on the underlying parameter values. As the parameters in our model are chosen in a way to describe a physiological situation the trivial steady state is unstable and will not be reached. However, there are scenarios where the *β*‐cells in the model eventually die out. This could be due to an abnormally high apoptosis rate *p*_4_ or artificially holding of the glucose concentration below the threshold value Ĝ by external insulin infusion, for example. While a reduction of the replication rate due to hypoglycemia is shown in [11] a complete disappearance of the *β*‐cell mass is an implausible result. The death of the organism would happen prior to the extinction of the *β*‐cells.

Our system allows for simulation of the glucose‐insulin regulatory system assuming *in vivo* situations. Our model builds a theoretical basis for description and explanation of dynamics derived from biological experiments. It supports the understanding of metabolic processes. Biological assumptions can be verified and quantification of data and parameters can be achieved. Additionally, the model promotes understanding of the interplay of the three different regulation feedback loops. The model is able to describe metabolic dynamics of the glucose‐insulin regulatory system also for the pathological case of type 1 or type 2 diabetes.

To illustrate one possible modification of the system a type 2 diabetes‐like simulation is done. Type 2 diabetes is characterized by insulin resistance of target cells, mainly muscle and fat cells. In consequence, these cells are not able to take up enough glucose from the blood. In this case the insulin sensitivity of the body cells is down‐regulated. To simulate this situation the model parameter for insulin sensitivity *p*_8_ is decreased arbitrary from the value *p*_8_ = 360 × 10^−3^to ${\tilde{p}}_{8}=360\times 10^{-5}$ while the other parameters given in Table 1 stay the same. This modification corresponds to a lower reaction of the target cells to insulin and therefore a decreased uptake of glucose from the blood.

**Figure 7:**
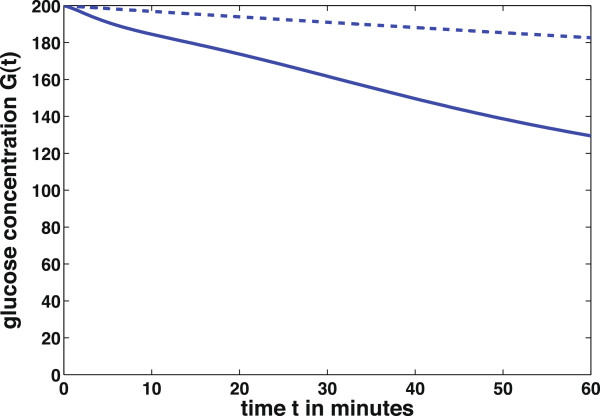
**Insulin resistance of target cells.** Blood glucose concentration at different values of insulin sensitivity *p*_8_. Physiological value *p*_8_ = 360 × 10^−3^(solid line) and pathological value ${\tilde{p}}_{8}=360\times 10^{-5}$ (dashed line).

Figure 7 shows that the blood glucose concentration *G* in the pathological case of insulin resistance (dashed line) decreases slower than in the physiological case (solid line). The body cells take up less glucose from the blood and therefore the hyperglycemia lasts longer in the pathological case.

There are also possibilities to simulate the regulatory system in a type 1 diabetes scenario. It can be done for example by increasing the apoptosis rate *p*_4_ in the cell cycle model. This results in dying *β*‐cells which is characteristic for this autoimmune disease. For a more detailed discussion about *β*‐cell mass in a type 1 diabetes scenario see Klinke [51]. There, *β*‐cell mass at onset of type 1 diabetes is concerned depending on body weight and the patient’s age.

In summary, our model is a basic approach to understand the processes within the glucose‐insulin regulatory system connected to the *β*‐cell cycle. It offers a wide range of possible modifications to incorporate further processes and can be adapted to many biological questions.

## Appendix

### Derivation of glucose dependent insulin storage dynamics

With time dependent glucose the major difference to the original model of Grodsky consists in time dependence of upper and lower integral bounds for *X*_1_ and *X*_2_:

$$\begin{array}{lll} \dot{X_{1}}(t) & =\frac{d}{\mathrm{dt}}\left(\int_{0}^{G(t)}\xi (\theta ,t)\text{dθ}\right),\; & \;\\ \dot{X_{2}}(t) & =\frac{d}{\mathrm{dt}}\left(\int_{G(t)}^{\infty }\xi (\theta ,t)\text{dθ}\right).\; & \; \end{array}$$

To determine these integrals, the differential equation for *ξ*(*θt*), given in [4], ‐ has to be solved first. Then this solution can be applied to the expressions for *X*_1_ and *X*_2_. In a final step we differentiate these terms with respect to time *t* to achieve differential equations $\dot{X_{1}}$ and $\dot{X_{2}}$.

### Solution of the differential equation $\dot{\xi }(\theta ,t)$

As only the packets with threshold *θ*below glucose concentration *G* are relevant for insulin secretion, two cases have to be distinguished. There is a differential equation for *θ* ≤ *G*(*t*),

(17) $$\dot{\xi }(\theta ,t)=-p_{9}\;\xi (\theta ,t)+f\xi_{0}(\theta )P(t)-\tilde{f}{\bar{X}}_{\text{max}}\xi (\theta ,t)+\tilde{f}\xi_{0}(\theta )X_{\infty }(t),$$

and for *θ* > *G*(*t*),

(18) $$\dot{\xi }(\theta ,t)=f\xi_{0}(\theta )P(t)-\tilde{f}{\bar{X}}_{\text{max}}\xi (\theta ,t)+\tilde{f}\xi_{0}(\theta )X_{\infty }(t),$$

with

$$\xi_{0}(\theta )=\xi (\theta ,0),\;X_{\infty }(t)=\int_{0}^{\infty }\xi (\theta^{\prime },t)d\theta^{\prime }=X_{1}(t)+X_{2}(t)\;\text{and}\;{\bar{X}}_{\text{max}}=\int_{0}^{\infty }\xi_{0}(\theta^{\prime })d\theta^{\prime }.$$

The only difference between the two equations is the term for insulin secretion, −*p*_9_*ξ*(*θ*,*t*), that occurs in the first but not in the second equation. Therefore, we restrict the following exploration to the case *θ* ≤ *G*(*t*).

The system that has to be solved is a linear non‐homogeneous ordinary differential equation for which there are standard solution methods like *variation of constants*. Using this method, the set of solutions for differential equation (17) is given as

$$\xi \left(\theta ,t\right)=\left\{e^{-(p_{9}+\tilde{f}{\bar{X}}_{\text{max}})t}\left(\left[\int_{0}^{t}e^{(p_{9}+\tilde{f}{\bar{X}}_{\text{max}})\tau }\left[\mathrm{fP}(\tau )+\tilde{f}X_{\infty }(\tau )\right]\xi_{0}(\theta )\mathrm{d\tau}+C\right],\;\right|\;C\in R\right\}$$

and for the differential equation (18) as

$$\xi (\theta ,t)=\left\{e^{-\tilde{f}{\bar{X}}_{\text{max}}t}\left(\left[\int_{0}^{t}e^{\tilde{f}{\bar{X}}_{\text{max}}\tau }\left[\mathrm{fP}(\tau )+\tilde{f}X_{\infty }(\tau )\right]\xi_{0}(\theta )\mathrm{d\tau}+C\right],\;\right|\;C\in R\right\}.$$

The constant *C* incorporates the initial conditions by *C* = *ξ*_0_(*θ*).

### Derivation of the differential equations $\dot{X_{1}}(t)$ and $\dot{X_{2}}(t)$

To derive a differential equation for the total amount of releasable insulin at glucose concentration *G* the expression

$$X_{1}(t)=\int_{0}^{G(t)}\xi (\theta ,t)\text{dθ}$$

has to be differentiated with respect to time *t*. Attaching the solution of $\dot{\xi }(\theta ,t)$ to *X*_1_ results in the following expression:

$$X_{1}\left(t\right)=e^{-(p_{9}+\tilde{f}{\bar{X}}_{\text{max}})t}\int_{0}^{G(t)}\xi_{0}\left(\theta \right)\text{dθ}\;\left[\int_{0}^{t}e^{(p_{9}+\tilde{f}{\bar{X}}_{\text{max}})\tau }\;\left[\mathrm{fP}(\tau )+\tilde{f}X_{\infty }(\tau )\right]\mathrm{d\tau}+1\right].$$

The differentiation of this equation with respect to time *t* then results in

$$\begin{array}{lll} \frac{d}{\mathrm{dt}}X_{1}(t)= & \dot{X_{1}}(t)=\frac{X_{1}(t)}{\int_{0}^{G(t)}\xi^{*}(\theta ,t)\text{dθ}}\xi^{*}(G(t),t)Ġ(t)-p_{9}X_{1}(t)\; & \;\\ & +\tilde{f}\int_{0}^{G(t)}\xi^{*}(\theta ,t)\text{dθ}[X_{1}(t)+X_{2}(t)]-\tilde{f}\int_{0}^{\infty }\xi^{*}(\theta ,t)\text{dθ}X_{1}(t)\; & \;\\ & +f\int_{0}^{G(t)}\xi^{*}(\theta ,t)\mathrm{d\theta P}(t),\; & \; \end{array}$$

with target distribution *ξ*^∗^(*θ*,*t*).

Analogously, we derive the differential equation for the total amount of non‐releasable insulin at glucose concentration *G*

$$X_{2}(t)=\int_{G(t)}^{\infty }\xi (\theta ,t)\mathrm{d\theta .}$$

The differential equation is given as

$$\begin{array}{lll} \frac{d}{\mathrm{dt}}X_{2}(t)=\; & \dot{X_{2}}(t)=\frac{-X_{2}(t)}{\int_{G(t)}^{\infty }\xi^{*}(\theta ,t)\text{dθ}}\xi^{*}(G(t),t)Ġ(t)\; & \;\\ & +\tilde{f}\int_{G(t)}^{\infty }\xi^{*}(\theta ,t)\text{dθ}[X_{1}(t)+X_{2}(t)]-\tilde{f}\int_{0}^{\infty }\xi^{*}(\theta ,t)\text{dθ}X_{2}(t)\; & \;\\ & +f\int_{G(t)}^{\infty }\xi^{*}(\theta ,t)\mathrm{d\theta P}(t),\; & \; \end{array}$$

with target distribution *ξ*^∗^(*θ*,*t*).

## Transition functions

The transition functions *u*_1_,…,*u*_4_ of the compartment model for insulin secretion are complex expressions with integrals over the target distribution in the insulin storage. With the concrete packet distribution given in [4] the integrals can be determined explicitly in terms of Hill functions:

$$\overset{G(t)}{\underset{0}{\int }}\xi^{*}\left(\theta ,t\right)\text{dθ}=\frac{X_{\text{max}}(t)\;G{\left(t\right)}^{k}}{C^{k}+G{\left(t\right)}^{k}}={\bar{X}}_{\text{max}}\frac{G_{1}(t)}{G_{1}(0)}\;\frac{G{\left(t\right)}^{k}}{C^{k}+G{\left(t\right)}^{k}},$$

and

$$\overset{\infty }{\underset{G(t)}{\int }}\xi^{*}\left(\theta ,t\right)\text{dθ}=\frac{X_{\text{max}}(t)\;C^{k}}{C^{k}+G{\left(t\right)}^{k}}={\bar{X}}_{\text{max}}\frac{G_{1}(t)}{G_{1}(0)}\;\frac{C^{k}}{C^{k}+G{\left(t\right)}^{k}}.$$

The parameters *f* and $\tilde{f}$ are proportionality factors. The constant character of these factors has to be changed in our case due to variable glucose and the connection to the *β*‐cell cycle that influences the maximum amount of insulin per pancreas. Therefore, $\tilde{f}$ is chosen glucose dependent and with a condition to ensure that

(19) $$X_{1}(t)+X_{2}(t)\leq X_{\text{max}}(t).$$

The function $\tilde{f}$ has the form

$$\tilde{f}\left(t\right)=-\frac{p_{9}\;f\;C^{k}\;G{\left(t\right)}^{h-k}}{{\bar{X}}_{\text{max}}\frac{G_{1}(t)}{G_{1}(0)}\left[\mathrm{fG}{\left(t\right)}^{h}(1+C^{k}\;G{\left(t\right)}^{-k})-p_{9}(P_{0}^{h}+G{\left(t\right)}^{h})\right]}.$$

This expression was found by analyzing condition (19) in steady state situation.

With these preliminaries the transition functions *u*_*i*_ can be given explicitly:

$$\begin{array}{ccc} u_{1}(t) & = & \frac{-p_{9}\;f\;G{\left(t\right)}^{h-k}\;C^{2k}}{\left[\mathrm{fG}{\left(t\right)}^{h}(1+C^{k}\;G{\left(t\right)}^{-k})-p_{9}(P_{0}^{h}+G{\left(t\right)}^{h})\right]\left[C^{k}+G{\left(t\right)}^{k}\right]},\;\\ u_{2}(t) & = & \frac{-p_{9}\;f\;G{\left(t\right)}^{h}\;C^{k}}{\left[\mathrm{fG}{\left(t\right)}^{h}(1+C^{k}\;G{\left(t\right)}^{-k})-p_{9}(P_{0}^{h}+G{\left(t\right)}^{h})\right]\left[C^{k}+G{\left(t\right)}^{k}\right]},\;\\ u_{3}(t) & = & f{\bar{X}}_{\text{max}}\frac{1}{G_{1}(0)}\;\frac{G{\left(t\right)}^{k}}{C^{k}+G{\left(t\right)}^{k}},\\ u_{4}(t) & = & f{\bar{X}}_{\text{max}}\frac{1}{G_{1}(0)}\;\frac{C^{k}}{C^{k}+G{\left(t\right)}^{k}}. \end{array}$$

These expressions were used for the simulations in Figures 5 and 6.

## Competing interests

The authors declare that they have no competing interests.

## Authors’ contributions

MG carried out the modeling, the mathematical analysis and wrote the manuscript. WzC contributed to the modeling process and supported the mathematical analysis. BAH supported the biological understanding of the problem and participated in the modeling process. CK supported the modeling process and the mathematical analysis of the model. All authors read and approved the final manuscript.
